# Rapid visualization of latent fingermarks using gold seed-mediated enhancement

**DOI:** 10.1186/s12951-016-0228-3

**Published:** 2016-11-25

**Authors:** Chia-Hao Su, Chun-Chieh Yu, Fong-Yu Cheng

**Affiliations:** 1 Institute for Translational Research in Biomedicine, Kaohsiung Chang Gung Memorial Hospital, Kaohsiung, 833 Taiwan; 2 Department of Biomedical Imaging and Radiological Sciences, National Yang Ming University, Taipei, 112 Taiwan; 3 Department of Chemistry, Chinese Culture University, 55, Hwa-Kang Road, Yang-Ming-Shan, Taipei, 11114 Taiwan

**Keywords:** Aptamers, Au nanoparticles, Au seeds, Fingermarks, Lysozyme

## Abstract

**Background:**

Fingermarks are one of the most important and useful forms of physical evidence in forensic investigations. However, latent fingermarks are not directly visible, but can be visualized due to the presence of other residues (such as inorganic salts, proteins, polypeptides, enzymes and human metabolites) which can be detected or recognized through various strategies. Convenient and rapid techniques are still needed to provide obvious contrast between the background and the fingermark ridges and to then visualize latent fingermark with a high degree of selectivity and sensitivity.

**Results:**

In this work, lysozyme-binding aptamer-conjugated Au nanoparticles (NPs) are used to recognize and target lysozyme in the fingermark ridges, and Au^+^-complex solution is used as a growth agent to reduce Au^+^ from Au^+^ to Au^0^ on the surface of the Au NPs. Distinct fingermark patterns were visualized on a range of professional forensic within 3 min; the resulting images could be observed by the naked eye without background interference. The entire processes from fingermark collection to visualization only entails two steps and can be completed in less than 10 min. The proposed method provides cost and time savings over current fingermark visualization methods.

**Conclusions:**

We report a simple, inexpensive, and fast method for the rapid visualization of latent fingermarks on the non-porous substrates using Au seed-mediated enhancement. Au seed-mediated enhancement is used to achieve the rapid visualization of latent fingermarks on non-porous substrates by the naked eye without the use of expensive or sophisticated instruments. The proposed approach offers faster detection and visualization of latent fingermarks than existing methods. The proposed method is expected to increase detection efficiency for latent fingermarks and reduce time requirements and costs for forensic investigations.

## Background

Fingermarks are deposited when the ridged skin surface of a finger touches an object and creates an imprint on the object’s surface [1, 2]. The unique patterns of fingermarks make them become one a key form of physical evidence in forensic investigations [2–4]. When imprinted on opaque media such as paint, fingermarks are directly visible to the naked eye. However, latent fingermarks are not visible to the naked eye and can only be detected through visualizing certain residues (such as amino acids and lipids) present within the fingermark [5–7]. The rapid and reliable visualization of latent fingermarks is important to help police quickly identify potential suspects by comparing crime-scene fingermarks against existing fingermark databases. Currently common methods include the use of carbon powder, cyanoacrylate or triketohydrindene hydrate. Carbon powder physically adsorbs the latent fingermarks, while cyanoacrylate and triketohydrindene hydrate react with the protein’s primary amine to visualize latent fingermarks. Recently, various optical, chemical and physical techniques based on nanomaterials have been developed to provide obvious contrast between the background and the fingerprint ridges [8–10].

Recently, studies of specific biomolecule-targeting and nanomaterials have raised the possibility of increasing selectivity and sensitivity in fingermark [11–13]. Wood et al. reported a highly selective technique using a lysozyme targeting-aptamer-based reagent to visualize latent fingermarks with fluorescence images [14]. Shan et al. [15] and Xu et al. [16] respectively combined electrochemistry with surface plasmon resonance (SPR) and enzyme immunoassay to detect fingermarks. Li et al. used the SPR of aptamer-tagged Au nanoparticles (NPs) to visualize fingermarks [17, 18]. Very recently, He et al. developed a simple method termed immunological multimetal deposition (iMMD) which combines immunoassay and conventional MMD to allow for the naked-eye visualization of sweat fingermarks using silver staining [19]. The iMMD method requires fewer steps and less time than conventional and improved MMD. However, background interference can negatively impact the visual enhancement of the fingermark ridges during the silver staining processes. In practice, the concentrations and incubation times of silver staining are difficult to calculate and control because of the unknown quantity of Au NPs in the fingermarks. For the silver staining solution, increased incubation time will produce an obvious background, thus reducing the visual contrast of the ridges.

**Scheme 1:**
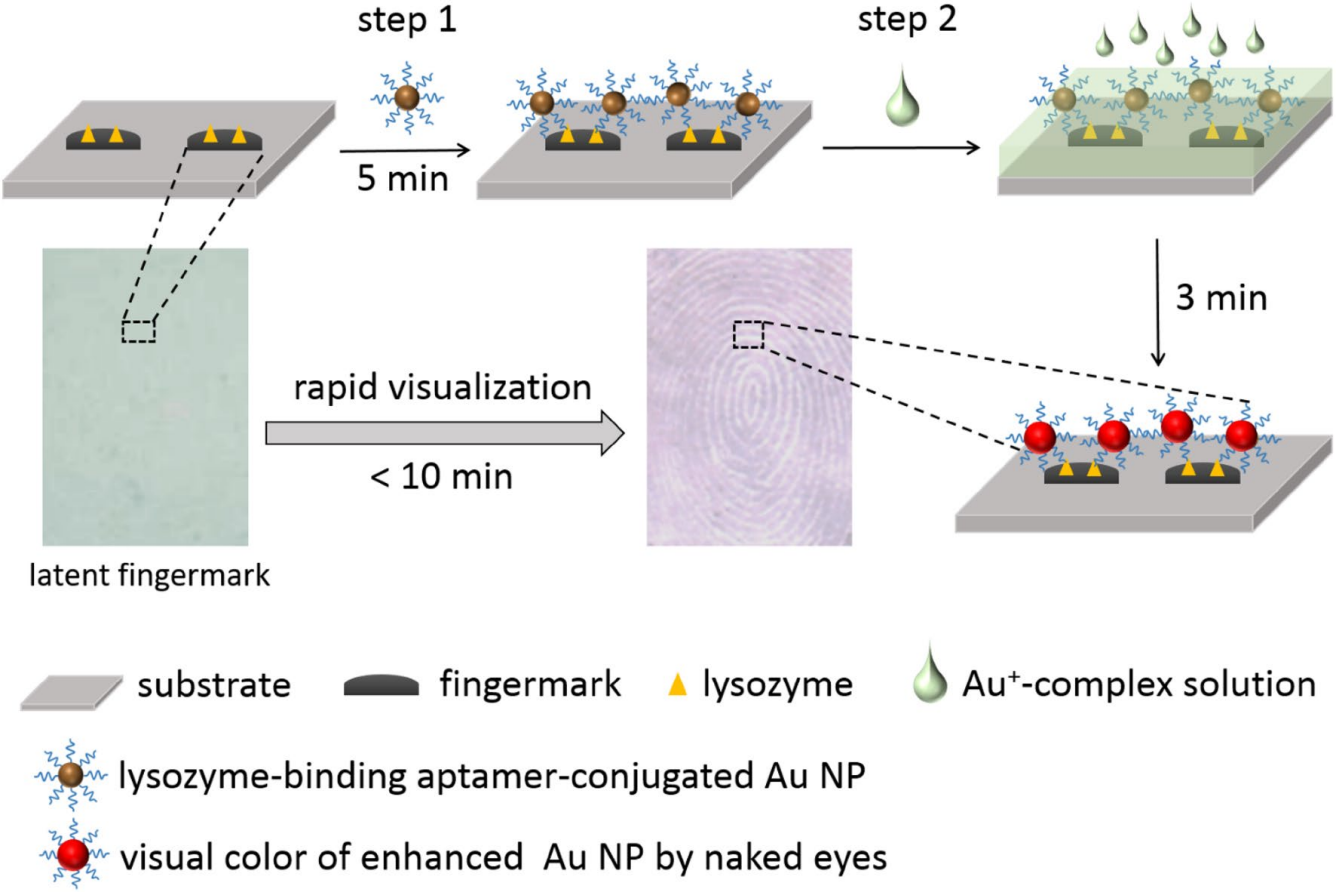
Schematic representation of rapid visualization of latent fingermarks by Au seed-mediated enhancement technique. Lysozyme-binding aptamer-conjugated Au NPs specifically recognize and target lysozyme in latent fingermarks (*step 1*). After treating with Au^+^-complex solution (*step 2*), bigger Au NPs with visible color are produced from Au seeds and can be clearly seen with the naked eye within 3 min. The total process from fingermarks collection to visualization can be completed within 10 min

To reduce the impact of background interference in techniques such as silver-staining, and to improve on the specific targeting of fingermarks in other methods based on the use of carbon powders, cyanoacrylate or triketohydrindene, this paper combines aptamer and metal-staining strategies [14, 20–22] for the rapid naked-eye visualization of latent fingermarks using a Au seed-mediated growth technique. In this technique, Au seed-mediated growth is a highly efficient chemical strategy for the synthesis of monodispersed nanoparticles. Au seed is as a nucleation center and can catalyze the reduction of Au^+^ ions at room temperature without the use of reducing agents. This reduction from Au^+^ to Au^0^ is an autocatalytic process and takes place on the surface of Au seeds. Scheme 1 shows a schematic representation of the proposed method. The process entails two main steps, and takes less than 10 min in total. We chose lysozyme as the targeted molecule because it is found in abundance in fingermark residues [20–22]. For the sensitive and selective targeting of lysozyme, Au seeds were conjugated with lysozyme-binding aptamers (LBA, 5′-thiol (SH)-TTTTTTATCAGGGCTAAAGAGTGCAGAGTTACTTAG-3′) [17, 18] by a covalent bond (Au–S bond). Thus, LBA-conjugated Au (LBA-Au) seeds can specifically recognize and target lysozyme in ridges. When LBA-Au seeds are treated with Au^+^-complex solution, concentrations of Au^+^ ions could be rapidly reduced and grown on the seed surface, resulting in larger Au NPs through a self-catalysis process (from Au^+^ to Au^0^) of the Au seeds. The large Au NPs are red in color, thus the unique patterns of latent fingerprints are rapidly visualized and can be observed by the naked eye in a short time (i.e., less than 10 min).

## Methods

### Preparation of tetrakis(hydroxypropyl)phosphonium chloride-stabilized Au (THPC-Au) NPs

THPC-Au NPs were prepared as previously described [23]. First, the THPC solution was prepared by diluting 12 µL of 80% THPC solution to 1 mL with deionized water. Subsequently, 1 mL of the as-prepared THPC solution, 0.5 mL of NaOH (1 M), and 45 mL of deionized water were vigorously stirred in a flask at room temperature for 5 min. 10 mL of HAuCl_4_ (5 mM) was then added. Once the color of the solution turned from light yellow to dark brown, indicating the formation of THPC-Au NPs, the mixture was stirred for another 2 h. THPC-Au NP solution was stored at 4 °C for further use. The average size of the as-prepared THPC-Au NPs was calculated using transmission electron microscopy (TEM, H-7500; Hitachi Koki Co., Tokyo, Japan). Electron micrographs of THPC-Au NPs and LBA-Au NPs were obtained by placing a drop of the sample onto a copper mesh coated with an amorphous carbon film and dried in a vacuum desiccator.

The particle concentration of THPC-Au NPs was based on the Au ion concentration measured using inductively-coupled plasma (ICP) analysis to calculate as µM. Au ion concentrations were measured by ICP in terms of mg/L and the concentration could be converted to mmole/L. The average size of THPC-Au NPs could then be determined via TEM imaging. We assumed Au NPs were spherical structures to calculate the volume of a single Au sphere, and the volume of a single Au atom could be calculated based on its diameter. Dividing the volume of one Au NP by the volume of one Au atom obtains the number of Au atoms contained in a single Au NP. We assumed that N Au atoms could form one Au NP. Therefore, the particle concentration (µM) of THPC-Au NPs could be calculated after dividing the Au ion concentration (µM) by N. The particle concentration of as-prepared THPC-Au NPs was 2.64 µM.

### Preparation of lysozyme-binding aptamer (LBA)-conjugated THPC-Au (LBA-Au) NPs

Thiol (SH)-modified LBAs (HS-LBA, 5′-HS-TTTTTTATCAGGGCTAAAGAGTGCAGAGTTACTTAG-3′) were used to prepare LBA-Au NPs. First, 26.4 µL of HS-LBA was mixed with 873.6 µL of deionized water, and then 100 μL of THPC-Au NPs (particle concentration: 2.64 µM) was added to the mixture and the solution was stirred for 4 h. The LBA-Au NPs (particle concentration: 0.264 µM) could then be directly used without further purification.

However, in experiments for the specific targeting and selection of lysozyme for LBA-Au NPs, Hex-LBA-Au NPs were prepared by replacing HS-LBA-Hex with HS-LBA. Hex-LBA-Au NPs were purified using a high-speed centrifuge at 100,000*g* for 10 min to remove free Hex-LBA in the supernatant. The precipitate was redispersed and stored in deionized water at 4 °C for further use.

### Preparation of Au^+^-complex solution

Cetyltrimethylammonium bromide (CTAB, 0.182 g) was dissolved in 9 mL of deionized water before adding 1 mL of HAuCl_4_ (5 mM). Subsequently, 60 µL of ascorbic acid (100 mM) was added to the mixture. The color of the solution changing from dark orange to colorless limpidity indicated the Au^+^-complex solution ([Au^+^] = 470 μM) was complete and ready for further use.

### Characterization

The absorption spectrum of the nanomaterials and biomolecules were determined using an ultraviolet–visible (UV–vis) spectrophotometer (HP8453; Agilent Technologies, Santa Clara, CA, USA).

### Test of Au seed-mediated enhancement

First, 997.5 µL of the Au^+^-complex solution was added to 1 mL of eppendorf, followed by 2.5 µL of THPC-Au NPs (particle concentration: 2.64 µM). Following the mixing of the Au^+^-complex solution and THPC-Au NPs, the seed growth reaction process was monitored using a UV–vis spectrophotometer at intervals of 10 s for 5 min.

### Collection of fingermarks

Professional-grade forensic tape was used to collect fingermarks. Volunteers first washed their fingers with soap and water and then dried them in air before pressing their fingertips on the tape. Fingermarks were collected immediately following deposition and after a 24 delay for further tests.

### Rapid visualization of latent fingermarks

First, 200 µL of LBA-Au NPs (particle concentration: 132 nM) were spread over the fingermarks on the substrate. After incubating for 5 min, the sample was rinsed two times with deionized water to remove free LBA-Au NPs. Next, 200–300 µL of the Au^+^-complex solution was spread over the fingermark region, followed by incubation for 2–3 min. Notably, the required visualization time of the latent fingermarks was based on the quantity of lysozyme from different volunteers. In our experimental tests, the time required to visualize latent fingermarks did not exceed 5 min.

## Results and discussion

**Fig. 1:**
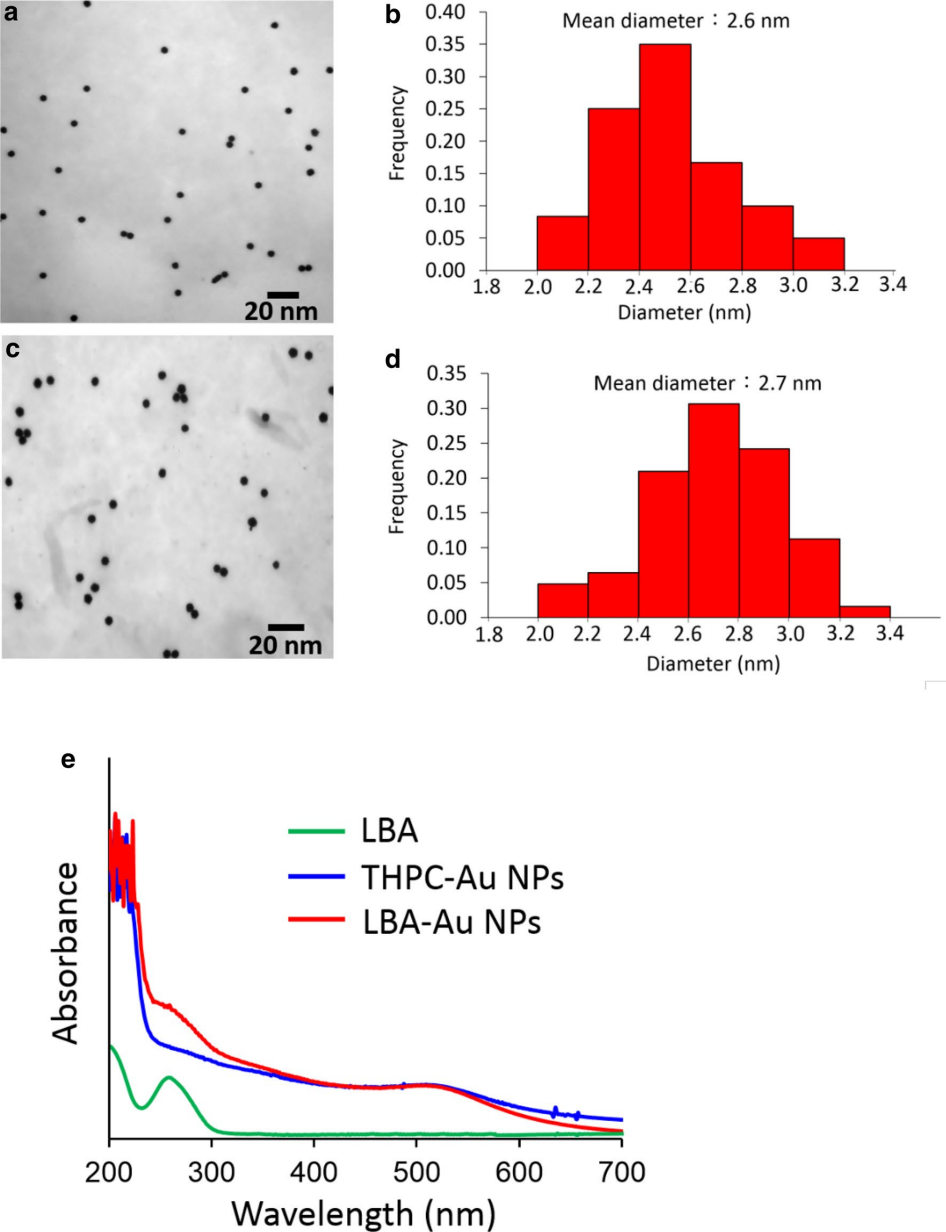
TEM images of **a** THPC-Au NPs and **c** LBA-Au NPs. Size distribution histograms of (**b**) THPC-Au NPs and **d** LBA-Au NPs. **e** Absorbance spectra obtained from LBAs, THPC-Au NPs, and LBA-Au NPs. The appearance of characteristic groups of LBAs and THPC-Au NPs observed in LBA-Au NPs supports the successful conjugation of LBAs on THPC-Au NPs

**Fig. 2:**
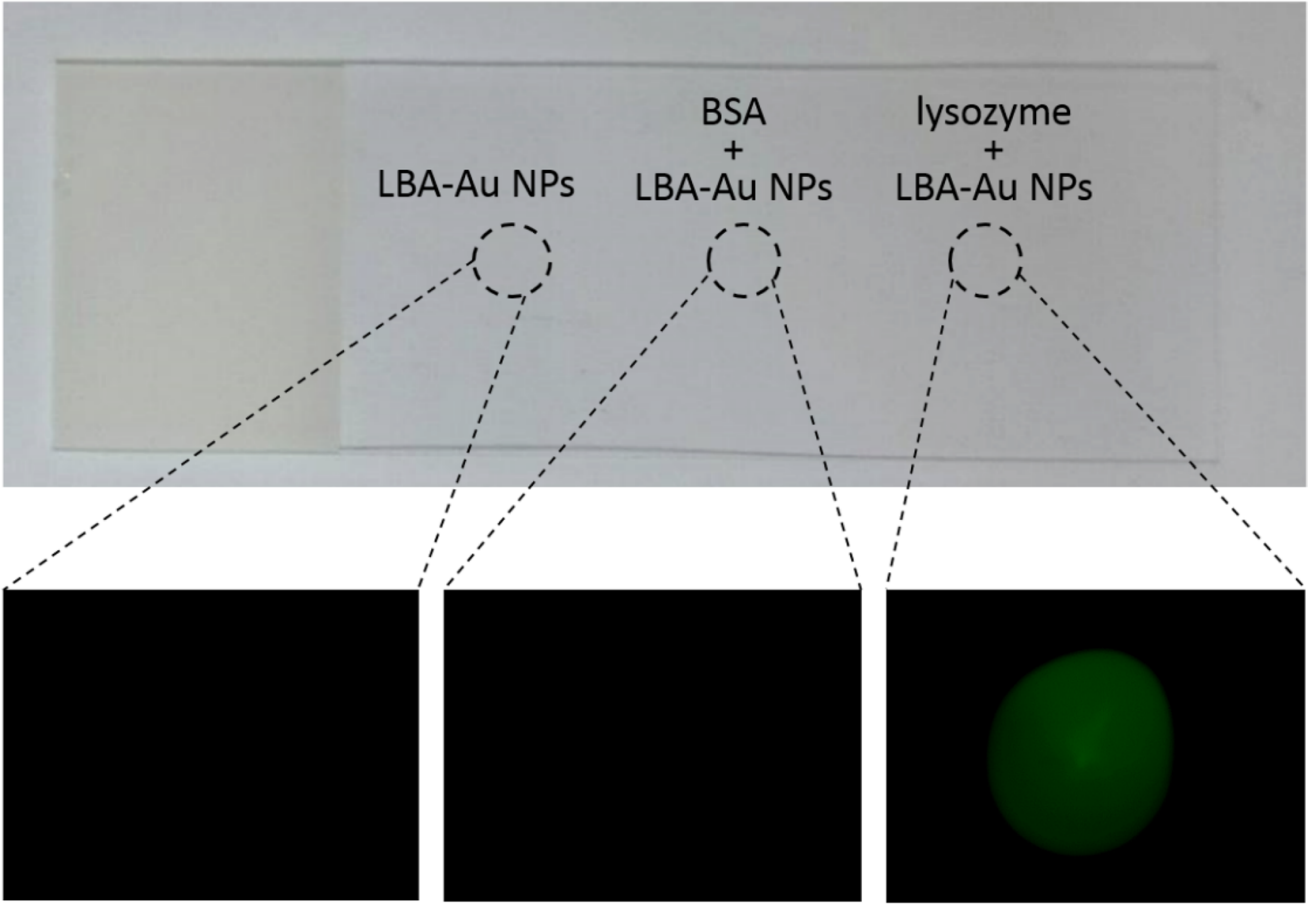
Demonstration of lysozyme selectivity of LBA-Au NPs by fluorescence imaging. To achieve the fluorescent function of LBA-Au NPs, both Hex- (fluorescent molecule) and thiol-modified LBAs (5′-H S-TTTTTTATCAGGGCTAAAGAGTGCAGAGTTACTTAG-Hex-3′) were used to replace thiol modified-LBAs in these experiments. The three regions defined by* black dotted circles* on the glass microscope slide (from *left to right*) were dropped with 10 µL of aqueous solution respectively containing 10 µM of PBS, bovine serum albumin (BSA), and lysozyme; the slide was then dried by air. Subsequently, the three regions were treated with LBA-Au NPs for 1 h, followed by washing with deionized water to remove non-binding LBA-Au NPs. Finally, the region in each *black dotted circle* was observed using fluorescence microscopy. The *left* region (PBS-treated region) exhibited no fluorescence after treatment with THPC-Au NPs, indicating that THPC-Au NPs had no non-specific targeting on the glass microscope slide. No fluorescence was observed in the* middle* region (BSA-existed region) after treatment with LBA-Au NPs because LBA-Au NPs could not bind to BSA and were thus removed during washing. The *right* region (lysozyme-existed region) had an obvious fluorescence after treatment with LBA-Au NPs because of the specific lysozyme-targeting of the LBA-Au NPs. These results indicated that LBA-Au NPs still had excellent selectivity for lysozyme after the LBAs were conjugated on the surface of the Au NPs

In this study, the tetrakis(hydroxypropyl)phosphonium chloride-stabilized Au (THPC-Au) NPs were chosen as Au seeds. THPC-Au NPs were prepared by the chemical reduction method described by Pham et al. [23]. The diameter of as-prepared THPC-Au NPs is ~2.6 nm. Subsequently, thiol-modified LBAs were conjugated with THPC-Au NPs using a covalent bond of Au–S. Figure 1a, b respectively show the transmission electron microscopy (TEM) images and size distribution histogram of THPC-Au NPs. Figure 1c, d respectively show the transmission electron microscopy (TEM) images and size distribution histogram of LBA-conjugated THPC-Au (LBA-Au) NPs. The TEM morphologies of THPC-Au NPs and LBA-Au NPs are identical, indicating that LBAs have no influence after being conjugated to the surface of THPC-Au NPs. Figure 1e shows the absorbance spectra of the LBAs, THPC-Au NPs, and LBA-Au NPs. LBAs have a characteristic absorption band at ~260 nm contributed from oligonucleotides. THPC-Au had a weak absorption band ~510 nm and the weak absorption is due to their smaller size. As seen in the spectrum of LBA-Au NPs, two characteristic bands of ~260 and ~510 nm are observed, indicating that LBAs are conjugated on the Au NPs. To confirm the lysozyme binding ability and selectivity of LBA-Au NPs, selective tests of lysozyme were designed on a glass microscope slide and monitored by fluorescence images (Fig. 2). Figure 2 shows the results of lysozyme-selection test of LBA-Au NPs. The PBS-treated region (the left region in Fig. 2) had no fluorescence after treatment with LBA-THPC-Au NPs. This indicates that LBA-THPC-Au NPs have no non-specific binding to the glass microscope slide. In the bovine serum albumin (BSA) region (the middle region in Fig. 2), no fluorescence was observed after treatment with LBA-Au NPs. This was due to LBA-Au NPs being unable to bind to BSA and they were later removed by washing with deionized water. As seen in lysozyme region (the right region in Fig. 2), after treatment with LBA-Au NPs for 5 min, fluorescence could be clearly observed. These tested results indicate that LBA-Au NPs could maintain the specific targeting function and selectivity of lysozyme after LBAs are conjugated on the surface of THPC-Au NPs.

**Fig. 3:**
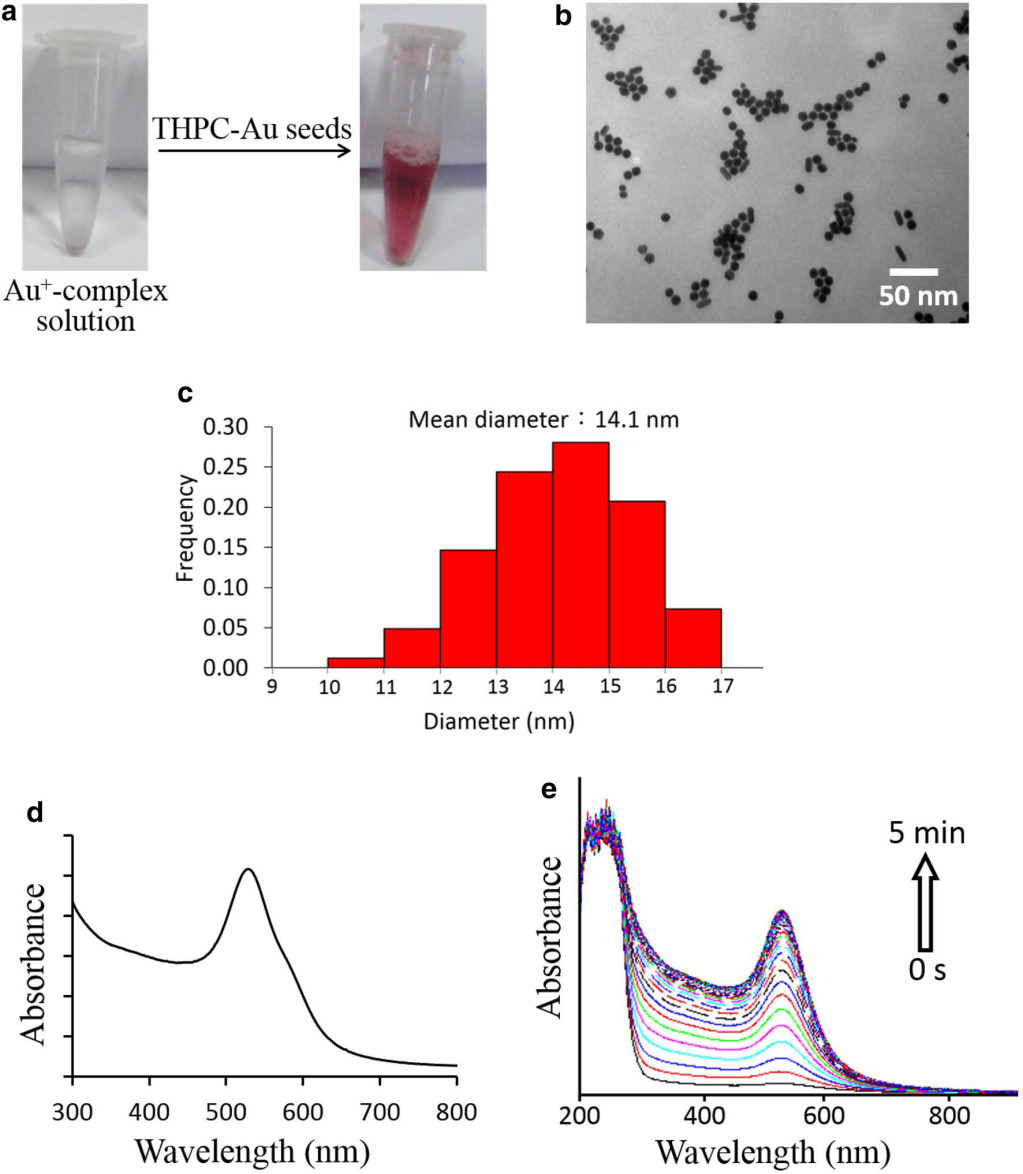
**a** Photographs of Au^+^-complex solution taken 1 min after the addition of THPC-Au seeds (final ratio in solution: [Au^+^]/[Au seed] = 152). Final [Au^+^] and [Au seed] in solution were 1 and 6.6 nM, respectively; **b** TEM image of resulting lager Au NPs after mixing Au^+^-complex solution and THPC-Au NPs for 1 min; **c** Size distribution histograms of LBA-Au NPs; **d** Absorption spectrum of the resulting lager Au NPs in Fig. 2b; **e** Evolution of the absorption spectrum of the solution after mixing Au^+^-complex solution and THPC-Au NPs, where the spectra were recorded at intervals of 10 s. Experimental conditions were the same as in Fig. 2a

To achieve the Au seed-mediated visualization of latent fingermarks, Au^+^-complex solution also played an essential role. The Au^+^-complex solution was composed of gold(III) chloride hydrate (HAuCl_4_), cetyltrimethylammonium bromide (CTAB), and ascorbic acid (AA). The preparation of the Au^+^-complex solution involves two steps. The first step mixes HAuCl_4_ and CTAB in the aqueous solution to produce Au^3+^-CTAB complexes [24–27]. The second step adds AA to reduce Au^3+^-CTAB complexes to Au^+^-CTAB complexes. In the presence of CTAB, AA cannot directly reduce Au^3+^ to Au^0^ [28, 29]. Importantly, the Au^+^-CTAB complexes only can by stable in an aqueous solution without Au seeds. Because Au^+^-CTAB complexes can be rapidly reduced to Au^0^ in the presence of Au seeds in a reduction reaction which does not require an additional reducing agent [24–29]. The reduction process of Au^+^-CTAB complexes occurred on the Au seed surface and the reduced Au^0^ directly grew on the seed surface resulting in particle growth of Au seeds, rapidly producing larger sized and visually colourful Au NPs. Thus, this Au seed-mediated enhancement system is very sensitive to the presence of Au seeds in the solution. Figure 3a shows the tested results of Au seed-mediated growth for THPC-Au NPs incubated with Au^+^-complex solution with a ratio ([Au^+^]/[Au seeds]) of 152. The color of solution changed from limpid to bright red within 1 min after incubation with THPC-Au NPs and the Au^+^-complex solution. This color change indicated that the THPC-Au seeds became larger Au NPs through a seed growth process. Figure 3b, c respectively show the TEM image and size distribution histogram of the resulting Au NPs with an average diameter of 14.1 nm. The characteristic absorption band of the larger Au NPs is ~530 nm, which corresponds to particle size (Fig. 3d). Time-dependent spectra of the solution were recorded at 10 s intervals after mixing with Au^+^-complex solution and THPC-Au NPs (Fig. 3e). Results in Fig. 3e show the significantly increased intensities at a 530 nm wavelength every 10 s, indicating that the sustained growth from Au seeds to larger Au NPs occurred over the course of 5 min. Actually, the red color of the solution in Fig. 3a could be observed by the naked eye within 20 s after mixing with Au^+^-complex solution and THPC-Au NPs.

**Fig. 4:**
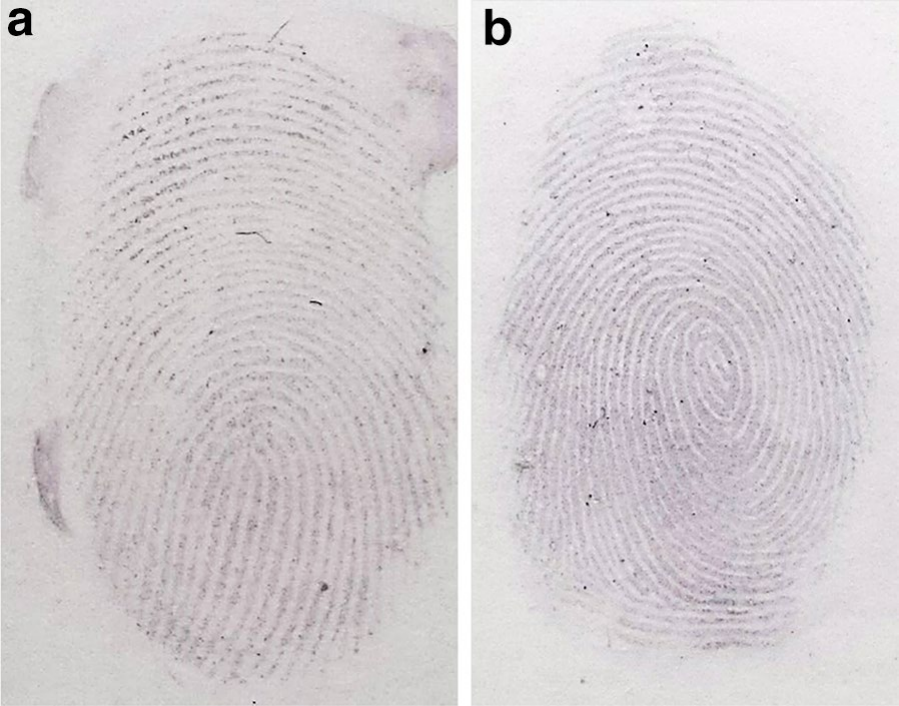
Optical images of latent fingermarks (**a**) collected immediately and **b** 24 h after deposition using professional forensic tape after treatment with Au seed-mediated enhancement. Both samples used the same experimental conditions. The treatment time of LBA-Au NPs (200 µL) and Au^+^-complex solution (300 µL) for fingermarks were 5 and 3 min, respectively

**Fig. 5:**
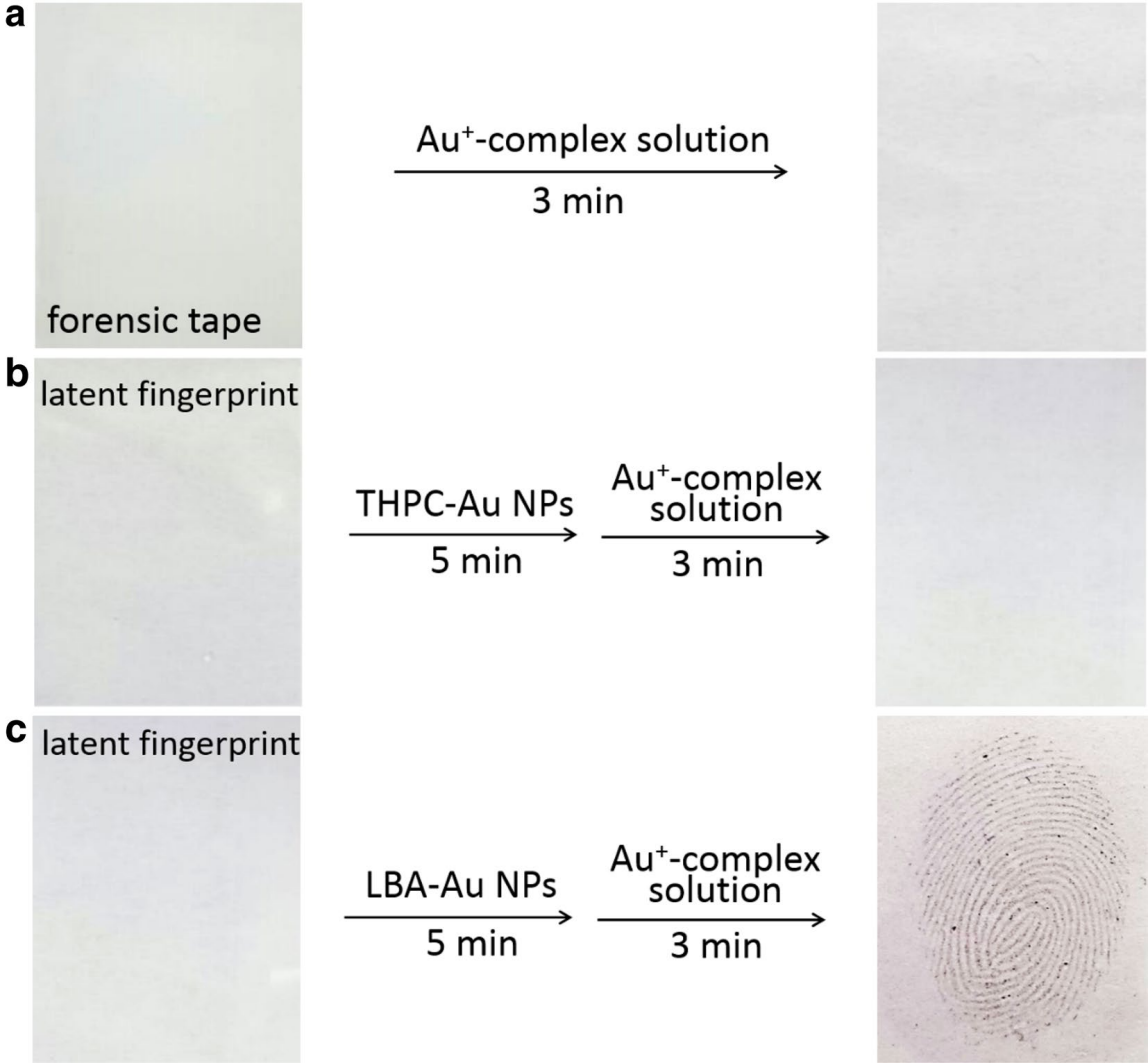
Collected latent fingermarks were separately visualized under three different conditions: **a** treated with Au^+^-complex solution alone; **b** incubated with THPC-Au NPs first and then treated with Au^+^-complex solution; **c** incubated with LBA-Au NPs first and then treated with Au^+^-complex solution. All latent fingermarks s were collected using professional-grade forensic tape. The used volumes of THPC-Au NPs and LBA-Au NPs were 200 μL with particle concentrations of 132 μM. The used volumes of Au^+^-complex solution were 300 μL

Figure 4 displays optical images of the rapid visualization of latent fingermarks collected using professional forensic tape after treatment with Au seed-mediated enhancement. Latent colourful collected immediately following deposition (Fig. 4a) and 24 h later (Fig. 4b) could be rapidly visualized and observed with the naked eye within 3 min. The ridge patterns of fingermarks are clear and no background interference is observed. The visual ridge patterns were due to the larger sized Au NPs generated from the LBA-Au seeds bound to lysozyme in fingermarks through a seed growth process. To exclude the possibility of a new generation of Au NPs being generated from the Au^+^-complex solution, the collected fingermarks were directly treated with Au^+^-complex solution for 3 min (Fig. 5a). The fingermarks could not be detected because of the lack of Au seeds, thus the Au^+^ of the Au^+^-CTAB complexes could not be reduced to Au^0^. To highlight the high Au seed-sensitivity in this method, THPC-Au NPs were designed to be replaced with LBA-Au NPs to first incubate latent fingermarks followed by treatment with the Au^+^-complex solution for 3 min (Fig. 5b). No fingermarks were visible on the tape because the THPC-Au NPs could not bind to lysozyme and were thus removed during the washing step, thus the Au^+^-CTAB complexes could not be reduced. However, when latent fingerprints collected using professional forensic tape (Fig. 5c) was first incubated with LBA-Au NPs and then treated with Au^+^-complex solution, fingermarks were clearly visualized within 3 min and could be observed with the naked eye when collected immediately or 24 h following deposition. This rapid visualization can significantly reduce the amount of time required for fingermark collection and further post-treatment. Importantly, the stickiness of professional forensic tape barely caused any non-specific adsorption of LBA-Au NPs and THPC-Au NPs, and valleys between fingermark ridges could be clearly observed in each test.

**Fig. 6:**
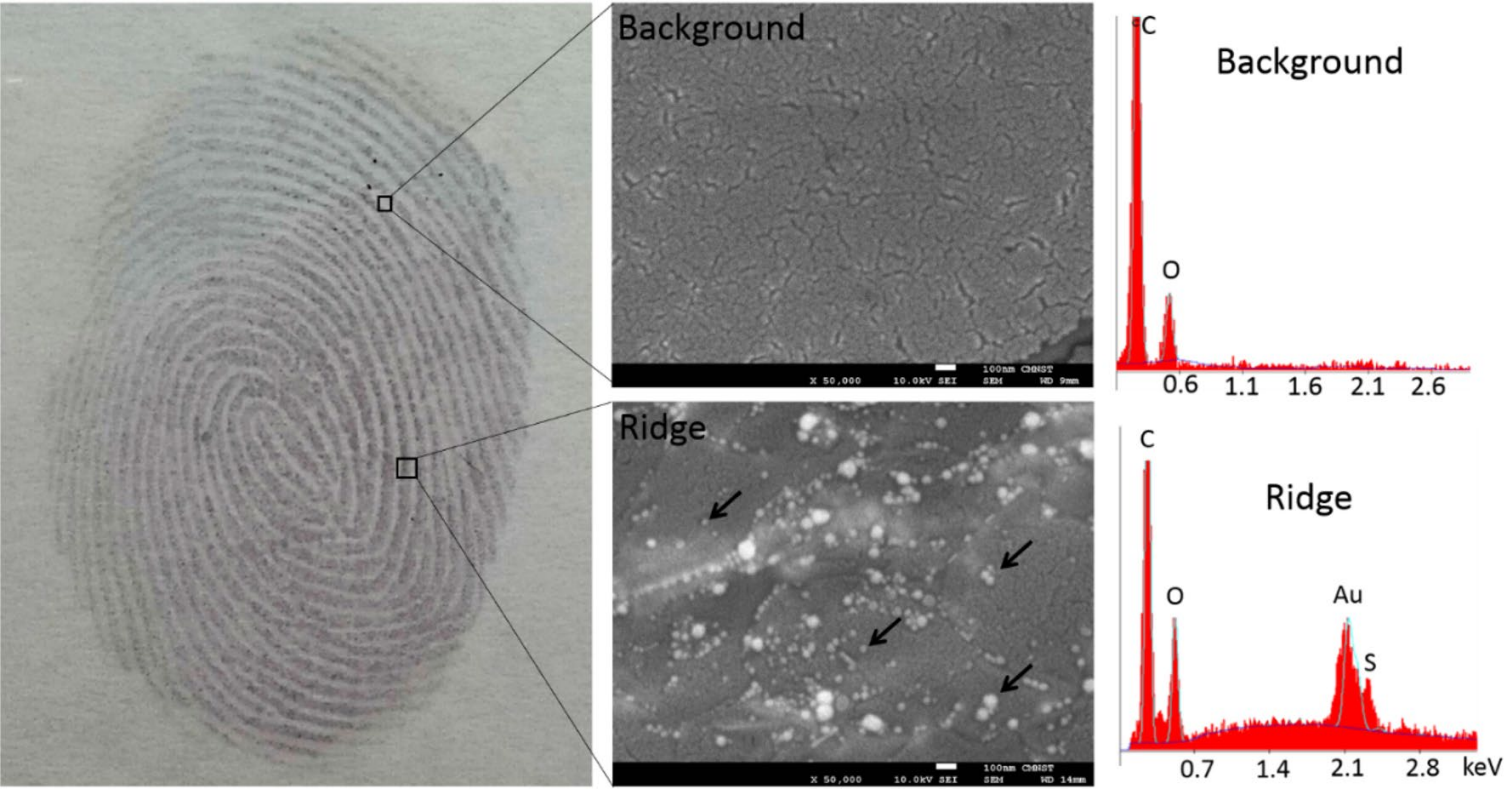
SEM images and EDX analyses of the ridges of fingermarks and the backgrounds between ridges after collected latent fingermarks were incubated with LBA-Au NPs first and then treated with Au^+^-complex solution. No signals of Au and Au NPs were detected and observed in the backgrounds between ridges of fingermarks, but Au NPs (*black arrows*) with different sizes appeared in the ridges of fingermarks and EDX proved their component was Au. These results indicated the visual ridges of fingermarks were certainly due to the growth of Au NPs from Au seeds. This latent fingermark was collected using professional-grade forensic tape. The used volumes of THPC-Au NPs and LBA-Au NPs were 200 µL with particle concentrations of 132 µM. The used volumes of Au^+^-complex solution were 300 µL. *Scale bars* in both SEM images are 100 nm

**Fig. 7:**
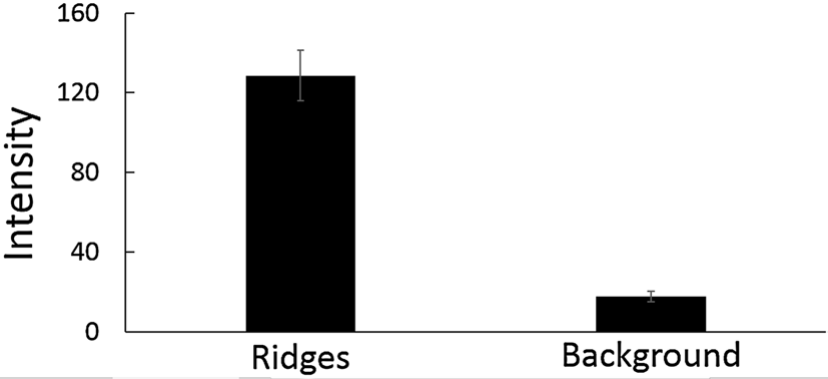
Quantitative analysis of latent fingermarks in Fig. 6 after treatment with Au seed-mediated enhancement. The intensity in the ridges and the backgrounds between ridges has an obvious difference and the p value is less than 0.001

**Fig. 8:**
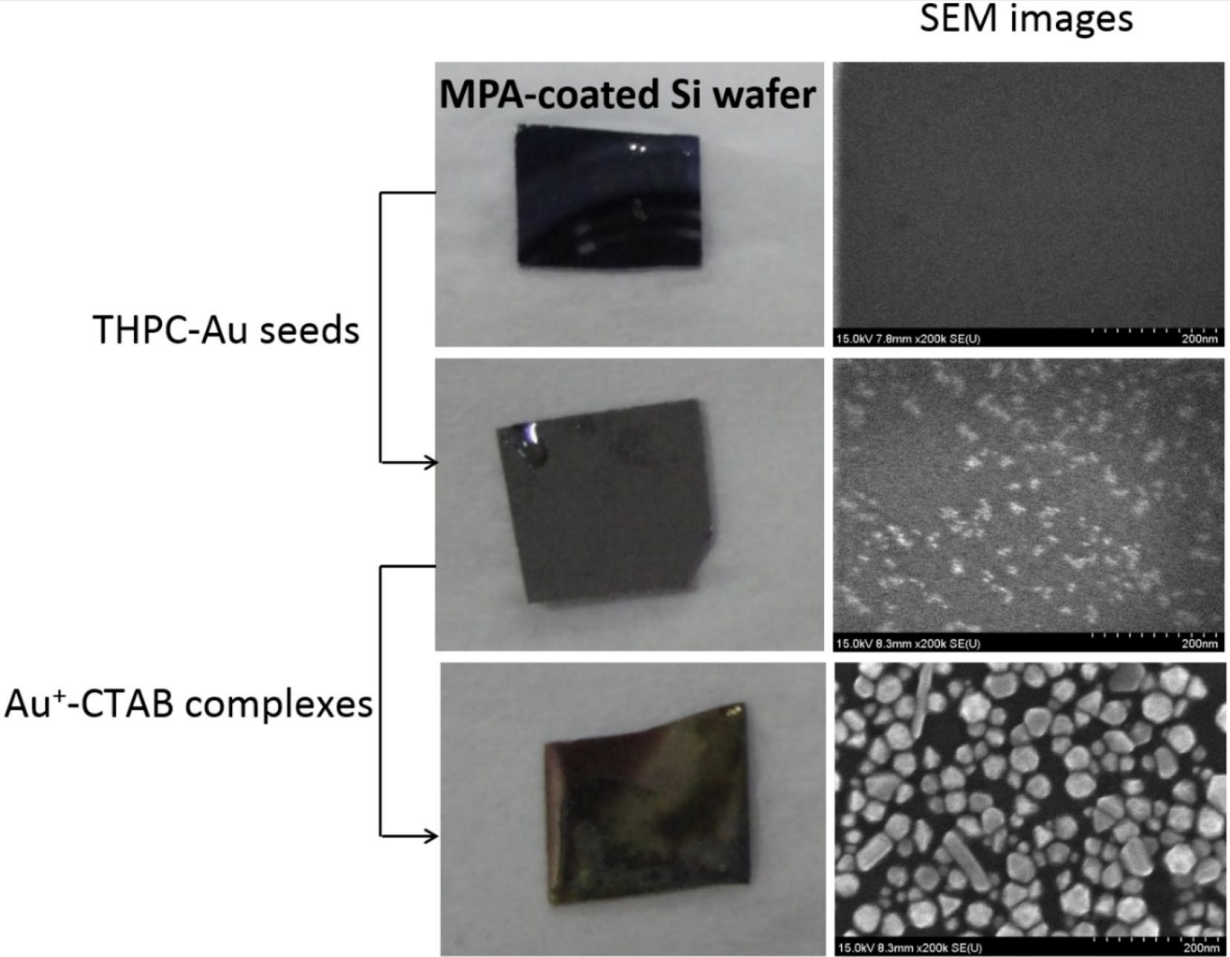
The optical and SEM images of MPA-coated Si wafer before and after treating with Au seed-mediated enhancement. THPC-Au seeds could be observed in SEM image of THPC-Au seed-treated Si wafer, but they were too small to see clearly. After THPC-Au seed-treated Si wafer treated with Au^+^-CTAB complexes, many Au NPs with different sizes produced on the surfaces of Si wafer. These results indicated the growth of Au NPs was certainly from Au seeds.* Scale bars* in all SEM images are 200 nm

To further prove the visual color of the ridges was due to the growth of Au NPs, the ridges of latent fingermarks and the backgrounds (valleys) between ridges were analysed by scanning electron microscopy (SEM) and energy-dispersive X-ray spectroscopy (EDX). Figure 6 shows no Au NPs or Au signals in the background, but many are observed in the ridges. The different sizes of the Au NPs were due to the self-catalysis reaction occurring in the solid–liquid interfaces. These results also indicate that LBA-Au seeds have no non-specific binding and adsorption on the backgrounds but rather only target lysozymes in the ridges. Figure 7 shows a quantitative analysis of latent fingerprints in Fig. 6 after Au seed-mediated enhancement. A clear difference is found in the intensity of the ridges and backgrounds and the p value is less than 0.001. The LBA-Au seed-targeted latent fingermarks are difficult to observe using SEM. To overcome this problem, we used 3-mercaptopropionic acid (MPA)-coated Si wafers to prove the growth process from Au seeds to NPs. An MPA-coated Si wafer was incubated with a high concentration of THPC-Au seeds for easy observation in SEM, and then THPC-Au seeds were conjugated on the surface of Si wafers using a Au–S bond, this is then followed by treatment with Au^+^-CTAB complexes. Figure 8 shows the SEM images of an MPA-coated Si wafer treated with Au seed-mediated enhancement. The SEM image of the THPC-Au seed-treated MPA-coated Si wafer shows the Au seeds, but their small size leaves them unclear. Following treatment with Au^+^-CTAB complexes, the growth process from Au seeds to NPs was very obvious and many larger Au NPs were produced on the Si wafer. We monitored the entire process from Au seed conjugation on the Si wafer surface to the growth of Au seeds to NPs. This process was repeated using professional forensic tape.

**Fig. 9:**
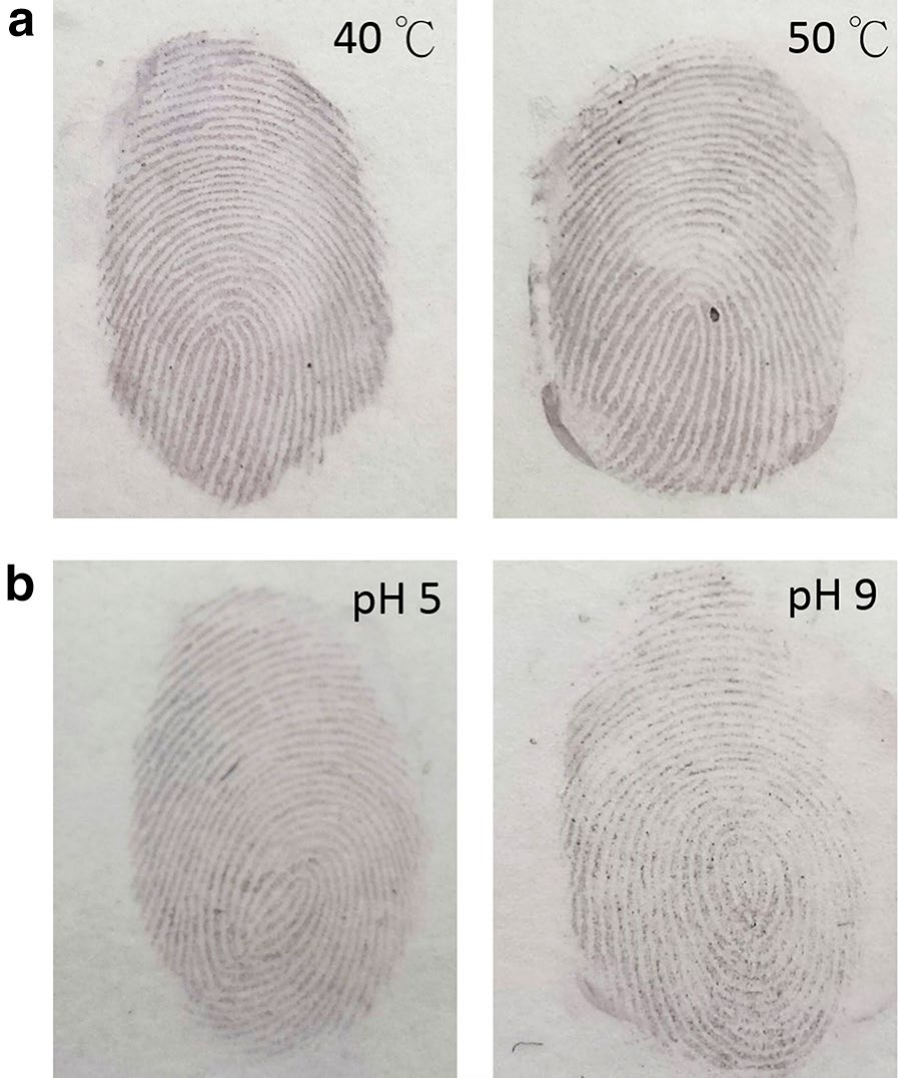
Optical images of latent fingermarks separately pre-treated with **a** temperature of 40 and 50 °C and **b** PBS at pH 5 and 9 for 24 h before treatment with Au seed-mediated enhancement. All samples used the same experimental conditions. The treatment time of LBA-Au NPs (200 µL) and Au^+^-complex solution (300 µL) for fingermarks were 5 and 3 min, respectively

Lysozyme is abundant in fingermark residues and is used here as a targeting biomolecule to visualize latent fingermarks. Although lysozyme is an enzyme, it can be stable in normal conditions. To demonstrate lysozyme tolerance under acidic and base solution conditions and at high temperatures, the collected latent fingermarks were pre-treated with PBS at pH 5 and 9 and at 40 and 50 °C before treatment with Au seed-mediated enhancement. Figure 9a shows the results of Au seed-mediated enhancement for the latent fingermarks after heating at 40 or 50 °C for 24 h. The ridges of fingermarks were still clear with no apparent damage. This indicates that lysozymes could be stable under temperatures of up to 50 °C. Figure 9b shows the results of Au seed-mediated enhancement for the latent fingermarks pre-incubated with PBS at pH 5 and 9 for 24 h. The ridges of fingermarks in both conditions (pH 5 and 9) were also clear, though the ridges of those prepared at pH 5 are relatively sharper, possibly because fewer lysozymes were removed by the pH 9 solution.

**Fig. 10:**
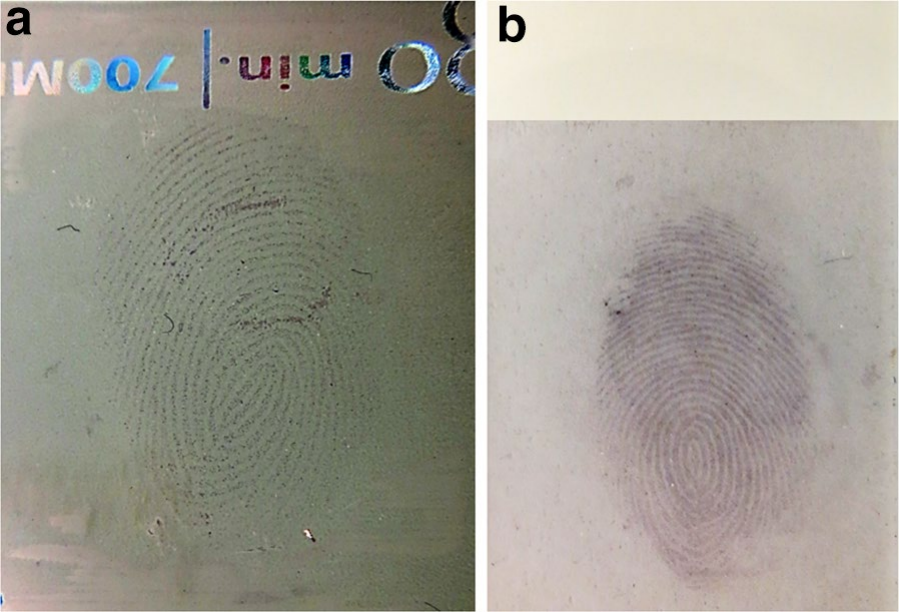
Optical images of using Au seed-mediated enhancement to visualize the latent fingermarks on **a** plastic and **b** glass. The plastic is the surface of commercial CD. The glass is general glass slide. Both samples used the same experimental conditions. The treatment time of LBA-Au NPs (200 µL) and Au^+^-complex solution (300 µL) for fingermarks were 5 and 3 min, respectively

In addition to using professional forensic tape, Fig. 10a, b respectively show the Au seed-mediated enhanced visualization of latent fingermarks using plastic and glass (see Fig. 10a). Ridge contrast intensity was found to be related to the use of different composites used in the CD surface of various commercial products. Au seed-mediated enhancement is not effective for visualizing latent fingermarks on porous substrates such as paper which adsorbs the aqueous working solution.

**Fig. 11:**
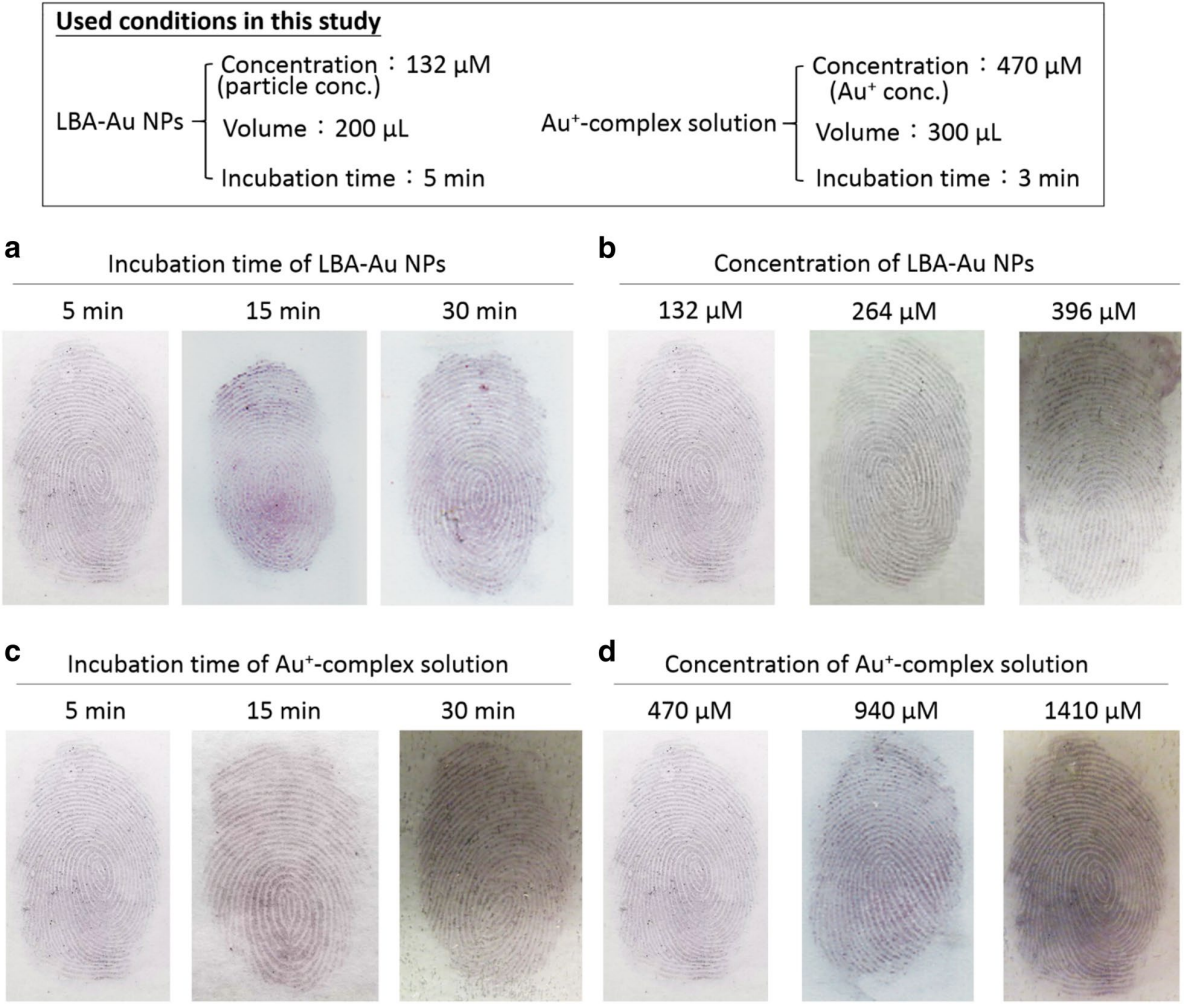
Optical images of using Au seed-mediated enhancement to visualize the latent fingermarks at different conditions with increasing: **a** the incubation time (5, 15, and 30 min) of LBA-Au NPs, **b** the concentration (132, 264, and 396 µL) of LBA-Au NPs, **c** the incubation time (5, 15, and 30 min) of Au^+^-complex solution and **d** the concentration (132, 264, and 396 µL) of Au^+^-complex solution. The upper frame means that the used conditions in this study. In **a**–**d** tests, only one parameter is changed in each test keeping the rest of the conditions analogous to the upper frame

The cyanoacrylate and triketohydrindene hydrate-based methods may fail to visualize latent fingermarks then the primary amine groups of proteins are completely consumed. In contrast, in Au seed-mediated enhancement, the growth of Au NPs can be easily sustained through the addition of more Au^+^-CTAB solution, allowing this method to work effectively even with smaller amounts of available proteins. The greatest difference between Au^+^-CTAB complexes and the silver staining solution used in iMMD is the image contrast between the ridges of fingermarks and background. For silver staining solution, longer incubation times will produce a significant background, reducing the visual contrast between the ridges and the underlying substrate. However, longer incubation using the Au^+^-CTAB complex produces a sharper image contrast between the ridges and the underlying substrate. The Au^+^-CTAB complexes only reacted with Au seeds and then reduced from Au^+^ to Au^0^ on the surface of the Au seeds. This self-catalysis process produced no background interference even with extended incubation times. In addition to incubation time, image contrast was also a function of solution concentrations. Using highly concentrated Au^+^ or Ag^+^ solutions might decrease the time required to enhance image contrast. However, too fast a reduction process can increase background interference. Using solutions in large volume could enhance the image contrast, but this requires additional time. Thus, both conditions (high concentrations and large volumes) easily produce background interference in the silver staining solution method, but no such interference occurs using the Au^+^-CTAB complex. Figure 11 shows fingermark images after treatment following long incubation times or high concentrations of the LBA-Au NPs or Au^+^-complex solutions. Increasing the LBA-Au NP incubation time from 5 min to 15 or 30 min (Fig. 11a), does not noticeably degrade the fingermark images because the originally used concentration (132 μM) of LBA-Au NPs is sufficient to bind specifically to fingermark lysosomes, and binding reaches saturation within 5 min, thus additional incubation time will not result in significant additional binding. Increasing the concentrations of LBA-Au NPs increased from 132 μM to 264 or 396 μM (Fig. 11b) also failed to significantly change the results. Treating latent fingermarks treated with 264 or 396 μM of LBA-Au NPs produced deeper colors, representing the additional Au content on the fingermarks. Another possible reason is that higher concentrations of LBA-Au NPs can further increase the binding amount of LBA-Au NPs on the lysozymes. Importantly, neither increased incubation time nor LBA-Au NP concentrations produced obvious background interference in Fig. 11a, b. Similarly, increasing the incubation time of Au^+^-complex solutions from 5 min to 15 or 30 min (Fig. 11c) still produced clear fingermark images with no obvious background interference. Increasing the concentration of Au^+^-complex solutions from 470 μM to 940 or 1410 μM (Fig. 11d) produces fingermark images with more pronounced color because Au^+^-complexs are consumptive substrates and can be reduced on the surface of the Au seeds until they are completely exhausted. Importantly, despite the pronounced fingermark colors in Fig. 11d, no obvious background interference is observed. These results clearly show that background interference is not increased by increasing the incubation time or the concentration of LBA-Au NPs or Au^+^-complex solutions in Au seed-mediated enhancement. All fingermarks are different, even those taken from different fingers from a single individual. In fact, the visualizing of latent fingermarks usually involves adding more solution or lengthening the incubation period until the marks appear on the substrates. In this situation, Au seed-mediated enhancement is more effective than the silver staining solution to obtain good image contrast without background interference.

## Conclusions

In conclusion, we report a simple, inexpensive, and fast method for the rapid visualization of latent fingermarks on the non-porous substrates using Au seed-mediated enhancement. LBA-Au NPs were used as Au seeds with a Au^+^-complex solution composed of HAuCl_4_, CTAB, and AA used as a growth agent. The Au^+^ ion of the Au^+^-CTAB complexes could be only reduced from Au^+^ to Au^0^ in the presence of the Au seeds. Importantly, the procedure requires only two main steps for latent fingermark visualization and can be completed in less than 10 min. In the first step, latent fingermarks are incubated with LBA-Au NPs for 5 min. In the second step, the latent fingermarks are treated with the Au^+^-CTAB complex solution for 3 min. In fact, latent fingermarks can be visualized for observation in less than 3 min after treatment with Au^+^-complex solution and without background interference. The proposed approach offers faster detection and visualization of latent fingermarks than existing methods. Moreover, the results are observable directly with the naked eye, without the use of expensive or sophisticated instruments. The proposed method is expected to increase detection efficiency for latent fingermarks, thus reducing time requirements and costs for forensic investigations and medical diagnostics.
